# Quality control in the diagnosis of *Trichuris trichiura* and *Ascaris lumbricoides* using the Kato-Katz technique: experience from three randomised controlled trials

**DOI:** 10.1186/s13071-015-0702-z

**Published:** 2015-02-05

**Authors:** Benjamin Speich, Said M Ali, Shaali M Ame, Marco Albonico, Jürg Utzinger, Jennifer Keiser

**Affiliations:** Department of Medical Parasitology and Infection Biology, Swiss Tropical and Public Health Institute, Basel, Switzerland; University of Basel, Basel, Switzerland; Laboratory Division, Public Health Laboratory-Ivo de Carneri, Chake Chake, Tanzania; Ivo de Carneri Foundation, Milan, Italy; Department of Epidemiology and Public Health, Swiss Tropical and Public Health Institute, Basel, Switzerland

**Keywords:** Soil-transmitted helminths, Diagnosis, Kato-Katz technique, Quality control, False-positive, Faecal egg counts, United Republic of Tanzania

## Abstract

**Background:**

An accurate diagnosis of soil-transmitted helminthiasis is important for individual patient management, for drug efficacy evaluation and for monitoring control programmes. The Kato-Katz technique is the most widely used method detecting soil-transmitted helminth eggs in faecal samples. However, detailed analyses of quality control, including false-positive and faecal egg count (FEC) estimates, have received little attention.

**Methods:**

Over a 3-year period, within the frame of a series of randomised controlled trials conducted in Pemba, United Republic of Tanzania, 10% of randomly selected Kato-Katz thick smears were re-read for *Trichuris trichiura* and *Ascaris lumbricoides* eggs. In case of discordant result (i.e. positive *versus* negative) the slides were re-examined a third time. A result was assumed to be false-positive or false-negative if the result from the initial reading did not agree with the quality control as well as the third reading. We also evaluated the general agreement in FECs between the first and second reading, according to internal and World Health Organization (WHO) guidelines.

**Results:**

From the 1,445 Kato-Katz thick smears subjected to quality control, 1,181 (81.7%) were positive for *T. trichiura* and 290 (20.1%) were positive for *A. lumbricoides*. During quality control, very low rates of false-positive results were observed; 0.35% (n = 5) for *T. trichiura* and 0.28% (n = 4) for *A. lumbricoides*. False-negative readings of Kato-Katz thick smears were obtained in 28 (1.94%) and 6 (0.42%) instances for *T. trichiura* and *A. lumbricoides*, respectively. A high frequency of discordant results in FECs was observed (i.e. 10.0-23.9% for *T. trichiura*, and 9.0-11.4% for *A. lumbricoides*).

**Conclusions:**

Our analyses show that the rate of false-positive diagnoses of soil-transmitted helminths is low. As the probability of false-positive results increases after examination of multiple stool samples from a single individual, the potential influence of false-positive results on epidemiological studies and anthelminthic drug efficacy studies should be determined. Existing WHO guidelines for quality control might be overambitious and might have to be revised, specifically with regard to handling disagreements in FECs.

## Background

The common soil-transmitted helminths (i.e. *Ascaris lumbricoides*, hookworm and *Trichuris trichiura*) affect approximately 1.5 billion people, cause considerable morbidity and account for an estimated 5.2 million disability adjusted life years (DALYs) [1,2]. An accurate diagnosis is important for the identification of infected individuals, for assessing anthelminthic drug efficacy, and for monitoring the control and elimination of soil-transmitted helminthiasis [3,4]. Currently, the most common diagnostic approach for soil-transmitted helminth infections in epidemiological studies is the copro-microscopic detection of helminth eggs using the Kato-Katz technique [5,6]. However, the Kato-Katz technique has limitations in terms of sensitivity, especially in low-intensity settings [3]. The sensitivity of the Kato-Katz technique is increased by analysing multiple thick smears from a single or, ideally, from multiple stool samples [7-15]. The specificity of the Kato-Katz technique is rarely investigated as studies examining the effect of multiple diagnostics usually merge all results to create a composite ‘gold’ standard, considering all positive results as “true positives” [16]. Henceforth, the possibility of false-positive results has been largely neglected and the frequency of false-positive is unknown [15]. However, assuming 100% specificity is unrealistic as false-positive results can arise, for example, when debris is confused as helminth eggs or by mistakes recording the data [16]. Tarafder *et al.* (2010) and Knopp *et al.* (2014) used Bayesian statistical methods to assess the specificity and sensitivity of the Kato-Katz technique for the diagnosis of soil-transmitted helminth infections [16,17].

In general, when a diagnostic method is used in an epidemiological study, a sub-sample (*e.g.* 10%) should be re-examined for quality control to ensure accuracy of the results [18]. However, two recent systematic reviews and meta-analyses revealed that rigorous quality control is the exception rather than the norm in epidemiological studies pertaining to soil-transmitted helminthiases [19,20]. A reason for that might be that, until recently, no guidelines were available for judging differences in faecal egg counts (FECs). This gap has been filled; in 2013 the World Health Organization (WHO) released a new guideline that offer some recommendations on how to perform quality control and how to handle differences in FECs [18]. Additionally, at the Swiss Tropical and Public Health Institute (Swiss TPH) an internal guideline has been recently developed. Of note, both of these guidelines distinguish between low and high infection intensities (see Table 1).

**Table 1:** **Two guidelines how to judge differences in faecal egg counts between the initial reading and the quality control reading of Kato-Katz thick smears**

**Guideline from the World Health Organization (WHO)**	**Internal guideline developed at the Swiss Tropical and Public Health Institute**
“If the expert identifies a difference in the egg count per gram of stool^b^ of more than 10% and more than four eggs, he or she should re-read the slide with the microscopist and discuss the reasons for the discrepancy”. ^a^ [18]	Results are considered as inconsistent if there is a difference in presence/absence of a specific helminth species, or if differences in egg counts exceed (i) 10 eggs for Kato-Katz thick smears with ≤100 eggs, or (ii) exceed 20% for Kato-Katz thick smears with more than 100 eggs.

^a^How to handle differences in presence/absence of helminth eggs is not explicitly stated in the WHO guideline. Therefore we assume that differences in presence/absence of helminth eggs do not require re-reading as long as the difference does not exceed 4 eggs.

^b^To calculate eggs per gram of stool, the egg counts from a single Kato-Katz thick smear are multiplied by a factor of 24. When multiplying egg counts by a factor of 24, differences in egg counts of less or equally to four eggs are not possible. Therefore we assume for this current work that the WHO aimed to apply their guideline for egg counts for Kato-Katz thick smears rather than per gram of stool (as it is indicated within a footnote of the WHO document).

In this study, we report new insights from detailed analysis of quality control data obtained over a 3-year period in a series of randomised controlled trials conducted in Pemba, United Republic of Tanzania. We assessed the frequency of false-positive results when using the Kato-Katz technique for the diagnosis of *T. trichiura* and *A. lumbricoides*. We also analysed the frequency of false-negative results and differences in FECs according to guidelines put forth by the WHO [18] and Swiss TPH.

## Methods

### Studies, subjects and quality control samples

In the years 2011, 2012 and 2013, we conducted three randomised controlled trials which evaluated new anthelminthic drugs or drug combinations against soil-transmitted helminths on Pemba Island, United Republic of Tanzania [21-23]. Children attending the schools in Wawi and Al-Sadik (both in 2011), Mchangamdogo and Shungi (both in 2012 and 2013) were invited to participate in the clinical trials. Within the frame of these randomised controlled trials, a total of 14,855 Kato-Katz thick smears were prepared and examined under a microscope at the Public Health Laboratory-Ivo de Carneri (PHL-IdC) by experienced laboratory technicians. Kato-Katz thick smears were prepared according to standard protocols. In brief, we used 41.7 mg templates, and the slides were read within 60 min to avoid over clearing of hookworm eggs [5,24,25]. Soil-transmitted helminth eggs were counted for each species separately (i.e. *A. lumbricoides*, hookworm and *T. trichiura*).

**Figure 1:**
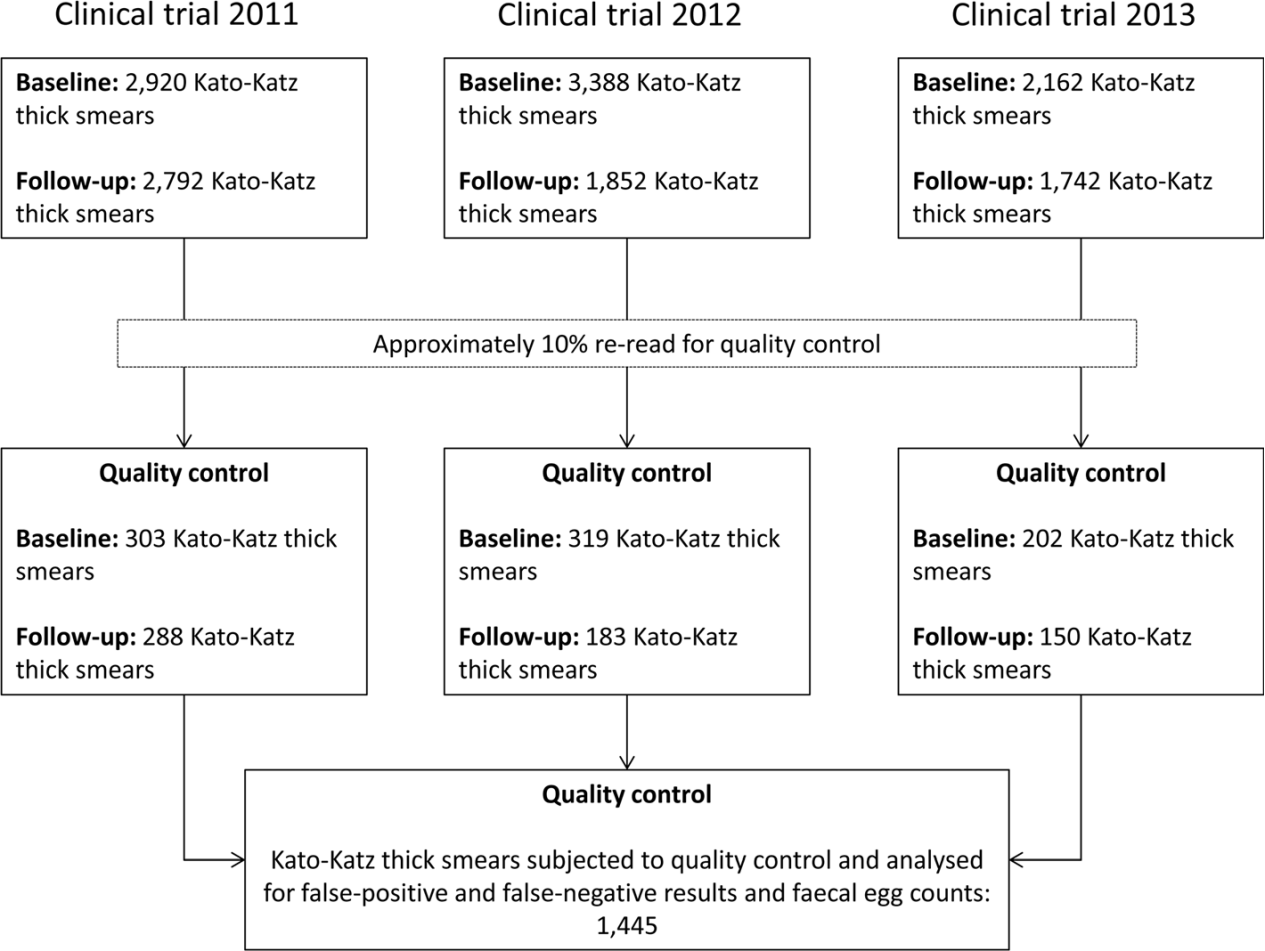
**Total number of Kato-Katz thick smears read in three randomised controlled trials conducted on Pemba Island, United Republic of Tanzania.** Flow chart detailing the number of Kato-Katz thick smears which were re-read for quality control, and hence were used for our analysis.

Figure 1 shows the number of Kato-Katz thick smears examined in each of the three trials before and after anthelminthic drug administration. Approximately 10% of the Kato-Katz thick smears were randomly selected and re-examined for quality control, on a day-to-day basis. As hookworm eggs disintegrate rapidly on Kato-Katz thick smears [24], quality control was restricted to *A. lumbricoides* and *T. trichiura*. Quality control was carried out within 24 hours after preparation of slides.

The results from the initial reading and from the subsequent quality control were compared. In case of discordant results (i.e. positive *versus* negative for a specific soil-transmitted helminth species and if the investigator judged the difference in FECs subjectively as too large) between the first two readers, a third microscopist was asked to re-examine the respective slide. Quality control (i.e. the second reading) was performed by a senior laboratory technician or by an investigator of the clinical trial. If a third reading was necessary, a third technician who did not previously examine the Kato-Katz thick smear was randomly chosen to re-examine the slide. The only exception was that in cases where a false-positive result was suspected, the first reader was asked to re-read the slide and show the observed egg to the investigator. All microscopists were blinded to previous results.

### Ethical considerations

For each of the three trials, ethical clearances from the cantonal ethics commission of Basel, Switzerland (EKBB) and from the Ministry of Health and Social Welfare of Zanzibar, United Republic of Tanzania were obtained [21-23]. The trials are registered at Current Controlled Trials (identifiers: ISRCTN08336605, ISRCTN54577342 and ISRCTN80245406). It was emphasised that study participation was voluntary and withdrawal possible at any time without further obligation. At the end of each study, all school-going children were offered albendazole (at a dose of 400 mg) according to national guidelines [26,27].

### Statistical analysis

Results were classified as false-positive if the original result was positive for a specific soil-transmitted helminth, but the results from the quality control (second reading), as well as from the third reading, were negative. A result was judged as false-negative if the original result was negative, but the quality control as well as the result from the third reading, were positive. We retrospectively calculated (i) the proportion of false-positive results on the overall number of samples; (ii) the proportion of false-positive results among the negative samples; (iii) the proportion of false-negative results among the overall number of samples; and (iv) the proportion of false-negative results among the positive samples. Species-specific differences among (ii) and (iv) were calculated with a two-sample test of proportion. FECs of confirmed false-positive and false-negative readings were descriptively analysed.

FECs from the primary reading were compared to the data from quality control according to two different guidelines put forward by WHO and Swiss TPH. Of note, at the time the clinical trials were implemented, no guidelines on judging FEC differences were available. In contrast to our in-house guideline, it is not explicitly stated in the WHO guideline how to address differences between the observed presence or absence of helminth eggs. Hence, we assumed that, according to WHO guideline, differences in presence/absence of helminth eggs do not require re-reading as long as the difference does not exceed the given range of 4 eggs (see Table 1). By comparing the initial readings to the results of the quality control, we retrospectively assessed the proportion of Kato-Katz thick smears which would have required a third re-reading according to the two different guidelines and which were therefore judged as discordant results. In addition, the proportion of agreeing Kato-Katz thick smears among the non-negative slides (i.e. FEC ≥ 1 either in the initial reading or the quality control) was calculated. These analyses were performed separately for *A. lumbricoides* and *T. trichiura*. All data were double entered into an Excel spreadsheet (Microsoft 2010) and cross-checked. Statistical analysis was conducted with Stata version 10.1 software (StataCorp.; College Station, TX, USA).

## Results

A total of 1,445 Kato-Katz thick smears (591 from the first, 502 from the second and 352 from the third trial), all selected at random, were subjected to quality control. The initial reading revealed 1,181 (81.7%) positive slides for *T. trichiura* and 290 (20.1%) positive slides for *A. lumbricoides.* As shown in Table 2, the quality control readings found five false-positive results for *T. trichiura* (0.35%) and four false-positive results for *A. lumbricoides* (0.28%). As the prevalence of *T. trichiura* was about four-fold higher than that of *A. lumbricoides*, there were fewer negative samples, which might have been reported as false-positive. The proportion of false-positive results for *T. trichiura* was higher than for *A. lumbricoides*; 1.89% and 0.35%, respectively. Hence, *T. trichiura* was significantly more often diagnosed as false-positive when analysing only the “true negative” results (p < 0.01).

**Table 2:** **Proportion of false-positive and false-negative results after a second quality control reading of Kato-Katz thick smears (in case of discordant results, slides were read a third time)**

	***Trichuris trichiura***	***Ascaris lumbricoides***
Total Kato-Katz thick smears read for quality control	1,445	1,445
Positive Kato-Katz thick smears	1,181	290
Negative Kato-Katz thick smears	264	1,155
Percentage of positive Kato-Katz thick smears	81.7%	20.1%
False-positive results^a^	5	4
Percentage of false-positive results	0.35%	0.28%
Percentage of false-positive results among negative Kato-Katz thick smears	1.89%^d^	0.35%^d^
Faecal egg counts of the false-positive Kato-Katz thick smears^b^	1, 3, 4, 7, 10	2, 2, 2, 12
False-negative results^c^	28	6
Percentage of false-negative results	1.94%	0.42%
Percentage of false-negative results among positive Kato-Katz thick smears	2.37%^e^	2.07%^e^
Faecal egg counts from the quality control of the false-negative Kato-Katz thick smears^b^	1, 1, 1, 1, 1, 1, 1, 1, 1, 2, 2, 2, 2, 3, 3, 3, 3, 3, 4, 4, 5, 6, 9, 10, 11, 12, 15, 16	1, 1, 1, 1, 6, 8

^a^Results were classified as false-positive if the original result was positive for a specific soil-transmitted helminth, but the results from the quality control (second reading), as well as from the third reading, were negative.

^b^Faecal egg counts per Kato-Katz thick smear.

^c^Results were classified as false-negative if the original result was negative, but the quality control as well as the result from the third reading, were positive.

^d^Significant difference (p < 0.01).

^e^No significant difference (p = 0.76).

Among the 1,445 re-examined slides, a total of 28 (1.94%) and 6 (0.42%) slides were detected as false-negative for *T. trichiura* and *A. lumbricoides*, respectively. The proportion of false-negative results among the positive Kato-Katz thick smears was 2.37% for *T. trichiura* and 2.07% for *A. lumbricoides*. No significant difference in proportion of false-negative results was detected between *T. trichiura* and *A. lumbricoides* (p = 0.76). FECs of the false-positive and false-negative results are listed in Table 2.

Differences in FECs between the initial reading and the quality control are presented in Table 3. From the 1,445 Kato-Katz thick smears read, discrepancies in FECs according to WHO guidelines [18] were detected in 345 (23.9%) and 148 (10.2%) of the Kato-Katz thick smears for *T. trichiura* and *A. lumbricoides*, respectively. According to the in-house guideline, differences in FECs were observed on 97 (6.7%) slides for *T. trichiura* and on 108 (7.5%) slides for *A. lumbricoides*. Additionally, differences in presence *versus* absence of FECs were observed in 51 (3.5%) and 32 (2.2%) slides for *T. trichiura* and *A. lumbricoides*, respectively. According to WHO guidelines, differences in presence *versus* absence were judged as acceptable as long as they did not exceed 4 eggs per slide, while our in-house guideline would recommend re-reading of the specific Kato-Katz thick smears (see Table 1). Hence, according to our internal guideline, a total of 145 (10.0%) *T. trichiura*-positive and 130 (9.0%) *A. lumbricoides*-positive slides would have been required to be re-read a third time (Table 3). When excluding all Kato-Katz thick smears which were negative in the initial as well as in the quality control reading, the percentage of disagreeing results increased considerably for both *A. lumbricoides* (i.e. 49.8% according to WHO guideline and 43.8% according to Swiss TPH guideline, Table 3) and for *T. trichiura* (i.e. 29.0% according to WHO guideline and 12.2% according to Swiss TPH guideline, Table 3). For both soil-transmitted helminth species, the WHO guideline would classify significantly more readings as discordant compared with our in-house guideline (p < 0.05).

**Table 3:** **Agreement in faecal egg counts (FECs) between initial reading and second quality control reading of Kato-Katz thick smears according to two different guidelines**

	**WHO guideline** ^**a**^		**Swiss TPH guideline** ^**a**^	
	***Trichuris trichiura***	***Ascaris lumbricoides***	***Trichuris trichiura***	***Ascaris lumbricoides***
Total number of Kato-Katz thick smears (positive/negative)	1,445 (1,189/256)	1,445 (297/1,148)	1,445 (1,189/256)	1,445 (297/1,148)
1. No. of Kato-Katz thick smears with difference in FECs (%)	345 (23.9%)	148 (10.2%)	97 (6.7%)	108 (7.5%)
2. No. of Kato-Katz thick smears with differences in presence/absence of helminth eggs (%)	51 (3.5%)	32 (2.2%)	51 (3.5%)	32 (2.2%)
3. No. of Kato-Katz thick smears with difference in FECs (%) among samples with egg counts	345 (29.0%)	148 (49.8%)	97 (8.2%)	108 (36.4%)
4. No. of Kato-Katz thick smears with differences in presence/absence of helminth eggs (%) among samples with egg counts	51 (4.3%)	32 (10.8%)	51 (4.3%)	32 (10.8%)
1. and/or 2. (%)	381 (26.4%)	164 (11.4%)	145 (10.0%)	130 (9.0%)
No. of Kato-Katz thick smears with discrepancies according to respective guideline^b^ (%)	345 (23.9%)	164 (11.4%)	145 (10.0%)	130 (9.0%)
3. and/or 4. and proportion as percentage (%) among Kato-Katz thick smears with positive egg counts	381 (32.0%)	164 (55.2%)	145 (12.2%)	130 (43.8%)
No. of Kato-Katz thick smears with discrepancies according to respective guideline^b^ (%) among Kato-Katz thick smears with positive egg counts	345 (29.0%)	148 (49.8%)	145 (12.2%)	130 (43.8%)

^a^See Table 1 for definition of the WHO and Swiss TPH guidelines.

^b^The WHO guideline state that differences which exceed 10% and more than four eggs require re-reading (see Table 1). However, in contrast to the Swiss TPH guideline, differences in presence/absence of helminth eggs are not explicitly stated. Therefore we assumed that differences in presence/absence of helminth eggs do not require re-reading as long as the difference does not exceed four eggs.

## Discussion

**Figure 2:**
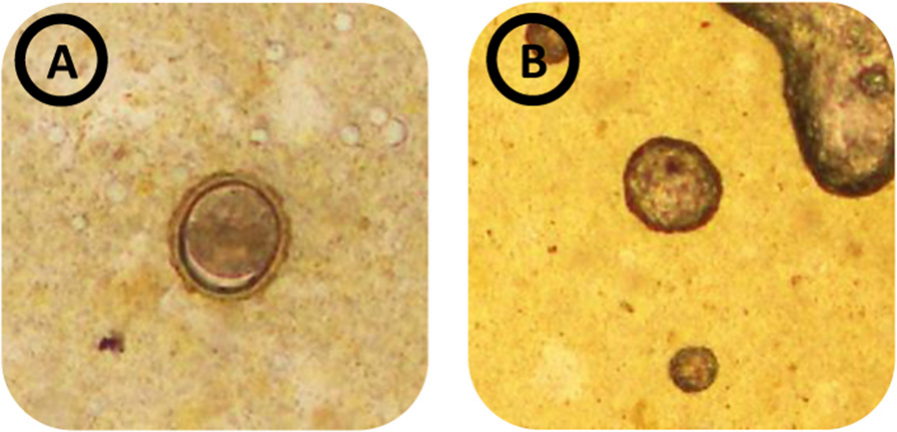
**Kato-Katz thick smears with an** ***Ascaris lumbricoides*** **egg (A), as well as debris that resembles an** ***A. lumbricoides*** **egg (B).**

The Kato-Katz technique is a widely used method for diagnosing soil-transmitted helminth infections in epidemiological surveys [14]. Usually, multiple Kato-Katz thick smears are examined per study participant to increase diagnostic sensitivity [7-15]. In general, it is assumed that an individual is positive if at least one out of several diagnostic tests revealed a positive result [16]. Thus far, the possibility of false-positive results in the diagnosis of soil-transmitted helminths was largely neglected. In this study we retrospectively calculated the frequency of false-positive diagnoses, facilitated by a detailed analysis of quality control results. We detected only low numbers of false-positive readings (*T. trichiura,* n = 5; *A. lumbricoides,* n = 4). When analysing only negative Kato-Katz thick smears, the proportion of false-positive results was significantly higher for *T. trichiura* (1.89%) than for *A. lumbricoides* (0.35%)*.* The fact that *T. trichiura* was more often over-diagnosed than *A. lumbricoides* might be explained by the smaller shape of its eggs, which might resemble debris in stool more closely. However, we hypothesise that within a single Kato-Katz thick smear, only a low number of debris particles can be confused with helminth eggs. Therefore, the false-positive Kato-Katz thick smears where several eggs were counted mistakenly (*e.g.* 10 or 12 eggs; Table 2), might point to another source of error (*e.g.* writing errors on the entry forms or eggs confused with eggs from different species [16,28]). Figure 2 shows some debris which might resembles an *A. lumbricoides* egg. Similar as for false-positive results, we suspect that the false-negative Kato-Katz thick smears which contained a larger number of eggs (Table 2) were probably due to writing errors. Another reason might be the tiredness of the readers due to a high number of Kato-Katz thick smears read per day. As a single Kato-Katz thick smear has low sensitivity, the proportion of false-negative diagnostics is usually assessed by examining stool samples with multiple diagnostic techniques, and by analysing multiple stool samples. As this was obviously not done within the current quality control investigation, the true number of false-negative diagnosed individuals is most likely underestimated [10,29].

Even though the proportion of false-positive was relatively low (i.e. 1.89% for *T. trichiura* and 0.35% for *A. lumbricoides*) its impact should not be neglected. While false-negative results can be corrected to some extent by examining multiple Kato-Katz thick smears, the probability of false-positive results increases as a function of examining multiple Kato-Katz thick smears [8]. Hence, in studies where multiple Kato-Katz thick smears are examined from each participant, the false-positive rate per individual might not be negligible. This hypothesis is confirmed by a recent study by Tarafder and colleagues (2010) who used Bayesian statistical methods to calculate the specificity of a single and multiple Kato-Katz thick smears. While they reported high specificity for a single Kato-Katz thick smear, specificity decreased by examining multiple Kato-Katz slides [16].

Differences in FECs between the initial and the quality control readings were assessed according to two different guidelines (Table 1). To our knowledge, we evaluated for the first time these guidelines with a large dataset from three randomised controlled trials. A relatively high frequency of discordant results was observed according to both guidelines. This observation indicates that an accurate counting of eggs in studies where high numbers of Kato-Katz thick smears are read in relatively short time frames is challenging. A particularly large number of disagreeing results was observed for the *A. lumbricoides*-positive slides. It is not entirely clear, whether *A. lumbricoides* eggs are generally more difficult to detect or if the high egg counts, which are more common for *A. lumbricoides*, were responsible for this result [30,31]. Given the fact that enumerating soil-transmitted helminth eggs is challenging, one might consider revising the current WHO guideline, which currently seem to be too firm and therefore might be overambitious. In our opinion another limitation of the WHO guideline is that a difference in presence *versus* absence of eggs is not considered a discordant result, as long as the FEC differed by not more than 4 eggs (see Table 1). However, verification of presence or absence of a helminth infection is crucial, as false-negative or false-positive diagnoses have important ramifications on patient management.

A limitation of our study is that we cannot evaluate the impact of further error sources of false-positive results, as for example sampling errors which can occur when children mix up or even share stool samples. Additionally, the aforementioned guidelines on how to judge and handle FEC differences in quality control had not yet been available at the time the clinical trials were conducted. Therefore, Kato-Katz thick smears with differing FECs were only re-read a third time if the investigator judged the difference in FECs as too large. This was done in a subjective way and not as strictly as might be suggested by the guidelines. Due to this reason, we did not have many third reading results for discordant FECs and could thus only compare the first and the quality control reading. It is important to note that discrepancies in those two readings could have equally arose due to reading errors by the initial microscopist as well as reading errors from the microscopist who conducted the quality control.

It should also be highlighted that our results are not directly transferable to other epidemiological settings. First of all, the technicians from the WHO Collaborating Centre PHL-IdC are highly skilled and they have examined tens of thousands of Kato-Katz thick smears over the past several years. Furthermore, the rigorous implementation of a quality control scheme might have increased the overall quality of the Kato-Katz thick smear readings leading to a low number of false-positive and false-negative readings. However, a high frequency of discordant FECs was observed, even though rigorous quality control was in place and technicians were highly experienced.

## Conclusion

We observed low rates of false-positive results of *A. lumbricoides* and *T. trichiura* when using the Kato-Katz technique. Our results indicate that especially in a setting where soil-transmitted helminth infections are highly prevalent, false-positive results will only have a small effect in overall prevalence estimates. However, in settings with low prevalence where it is challenging to identify infected individuals, false-positive results might influence treatment allocation, results of epidemiological studies and monitoring of control programmes. Examining multiple stool samples from participating individuals will further increase the frequency of false-positive results. Additionally, we have shown that an accurate counting of helminth eggs is a formidable challenge, even for highly skilled technicians. Therefore, the WHO guideline might be too strict and one might additionally cast doubt on the reliability of FECs as well as egg reduction rate, which has recently been proposed as the single most important metrics for assessing anthelminthic drug efficacy. We recommend to validate the current WHO guideline within different settings and to adapt it if the frequency of discordant results remains equally high.
